# Colorectal cancer pain upon diagnosis and after treatment: a cross-sectional comparison with healthy matched controls

**DOI:** 10.1007/s00520-022-06803-2

**Published:** 2022-01-13

**Authors:** Maria Lopez-Garzon, Paula Postigo-Martin, Ángela González-Santos, Manuel Arroyo-Morales, Alexander Achalandabaso-Ochoa, Antonio Manuel Férnández-Pérez, Irene Cantarero-Villanueva

**Affiliations:** 1 ‘Cuídate’ From Biomedical Group (BIO277), Instituto de Investigación Biosanitaria Ibs, University of Granada, Granada, Spain; 2 Department of Physiotherapy, Faculty of Health Sciences, University of Granada, Granada, Spain; 3 Instituto de Investigación Biosanitaria Ibs, GRANADA, Granada, Spain; 4 Unit of Excellence On Exercise and Health (UCEES), University of Granada, Granada, Spain

**Keywords:** Body composition, Cancer pain, Colorectal cancer, Muscle strength, Pain measurement

## Abstract

**Background:**

The current study sought to explore whether cancer pain (CP) already exists in patients at colorectal cancer (CRC) diagnosis before treatment compared with patients with colorectal cancer (CRC) after treatment and a healthy matched control group. The study also sought to examine whether factors related to physical health status could enhance pain processes.

**Methods:**

An observational cross-sectional study was conducted following the STROBE checklist. Twenty-nine newly diagnosed and forty post-treatment patients with CRC and 40 healthy age/sex-matched controls were included for comparison. Pain, local muscle function, and body composition outcomes were assessed by a physiotherapist with > 3 years of experience. ANCOVA and Kruskal–Wallis tests were performed, with Bonferroni and Dunn-Bonferroni post hoc analyses and Cohen’s *d* and Hedge’s effect size, as appropriate.

**Results:**

The analysis detected lower values of pressure pain threshold (PPT) points, the PPT index, and abdominal strength and higher values of self-reported abdominal pain in newly diagnosed patients, with even more marked results observed in the post-treatment patients, where lower lean mass and skeletal muscle index values were also found than those in the healthy matched controls (*p* < 0.05). In the post-treatment and healthy matched control groups, positive associations were observed between the PPT lumbar dominant side points and abdominal isometric strength and lean mass, and negative associations were observed between the lumbar dominant side points and body fat (*p* < 0.05).

**Conclusion:**

Upon diagnosis, patients with CRC already show signs of hyperalgesia and central sensitization and deteriorated physical conditions and body composition, and this state could be aggravated by subsequent treatments.

## Introduction

Cancer pain (CP) is one of the most prevalent and concerning aspects of the disease that patients with cancer must face, and it occurs in more than 60% of patients across all cancer stages [1], even from diagnosis [2]. This pain is very difficult to manage because it is a poorly understood and undertreated syndrome [3] that involves crucial health expenditures [4].

A systematic classification of chronic pain was developed by the International Association for the Study of Pain (IASP) that distinguishes chronic primary and chronic secondary pain syndromes. When pain persists or recurs for more than 3 months, it is considered chronic pain. In some conditions where pain may be considered a disease, the term chronic primary pain is used. However, in other cases, pain is secondary to an underlying disease, such as chronic cancer-related pain [5]. Additionally, the term central sensitization is defined by IASP as “increased responsiveness of nociceptive neurons in the central nervous system to their normal or subthreshold afferent input.” Clinically, sensitization may only be inferred indirectly from phenomena such as hyperalgesia or allodynia [6].

The presentation of chronic pain and central sensitization in patients with colorectal cancer (CRC) in the survival phase is well established [7, 8]. This abnormal processing of nociceptive inputs decreases the pressure pain threshold (PPT) [9]; therefore, low PPT in local and distant areas of cancer reflects primary hyperalgesia and central sensitization, which can increase perceived pain [10]. Depending on its pathogenesis, CP physiopathology may be of nociceptive, neuropathic, mixed, or psychogenic origin. After treatment, a state of central sensitization is increased in 75% of patients with CRC compared with that in healthy matched controls [8]. Among the possible factors influencing this state are cancer treatments, such as surgery [11], chemotherapy, and radiotherapy [8, 12, 13]; a state of prolonged nociceptive or neuropathic pain [14]; other factors related to muscle and adipose tissue that are closely related to CP [8, 15]; and certain behaviors in patients, such as kinesiophobia [16], which may increase pain perception.

In patients newly diagnosed with CRC who did not undergo cancer treatment, abdominal pain may already be present [2]. Tumors themselves induce CP by constricting or invading surrounding tissue, inducing infection or inflammation, or releasing chemicals. Tumor-induced visceral (nociceptive, neuropathic, or mixed) pain can also promote a central sensitization state [14]. However, the psychological distress of the impact of cancer diagnosis (which involves fear, anxiety, pain catastrophizing, and other responses) influences central sensitization and may modulate pain [17] by increasing the level of systemic inflammation through activation of the hypothalamic–pituitary–adrenal axis and sympathetic nervous system [18]. Additionally, these patients present factors related to unhealthy lifestyle habits that are risk factors for CRC appearance [19], which could also be factors that influence the early presentation of CP, as indicated in other populations [20, 21].

Although cancer treatment may induce pain, how this may be already established from the moment of diagnosis is unclear. Therefore, it would be interesting to fully elucidate this early CP appearance to offer tailored interventions to prevent or mitigate CP. Therefore, the current study sought to explore whether CP already exists in patients with CRC upon diagnosis before cancer treatment compared with patients after treatment and a healthy matched control group. The study also sought to examine whether factors related to the physical health status could influence pain processes.

## Methods

### Study design and participants

We conducted an observational cross-sectional study following the Strengthening the Reporting of Observational Studies in Epidemiology (STROBE) checklist [22]. For this study, the baseline evaluation of two cohorts (newly diagnosed *n* = 29; post-treatment *n* = 40) and 40 healthy age/sex-matched controls were included for comparison. Healthy age/gender-matched controls were recruited through announcements by the University of Granada on social networks. Both previous cohorts had the following inclusion criteria: (1) patients of legal age (> 18 years), (2) patients diagnosed with CRC (stage I to IIIa), (3) patients on a waiting list for surgery (newly diagnosed study), or (4) patients completed their medical treatment (post-treatment group). Patients with any medical contraindication or musculoskeletal condition to perform the assessments (e.g., chronic lumbar pain, fibromyalgia, or osteoarthritis), any abdominal surgery, or any previous cancer treatment (newly diagnosed group) were excluded. After the first contact, the patients were contacted by telephone for an appointment at the Sport and Health Research Center or Physiotherapy Laboratory of the Health Science Faculty of the University of Granada. All the participants signed an informed consent form before participating in the study.

The study protocols were approved by the Research Ethics Committee of the University of Granada (0572-M1–16 and 1087-N-16), and the study was performed in accordance with Law 14/2007 on Biomedical Research and the guidelines of the World Medical Association Declaration of Helsinki.

### Outcomes

The same evaluation protocols and assessment instruments (model and brand) were used in all the participants. Evaluations were made by a trained researcher with experience in the evaluation of patients with a CRC > 3 years. The patients were asked if they had taken any rescue analgesics in the last 24 h; if so, the assessment could be postponed. The demographic and clinical details were entered from the medical reports of the patients.

### Pain

#### Pressure pain thresholds (PPT) (kilopascals, kPA)

Testing was performed using an electronic algometer (Somedic AB. Farsta., Sweden) at the dominant and nondominant lumbar, supraumbilical, infraumbilical, and second metacarpal points, with a perpendicular diameter of 1 cm (absolute value). At each point, the evaluation was performed three times with a rest of 30 s, and progressive increases in force (30 kPA/s) were applied until the first perception of change from pressure to pain, which was previously explained to the participants. The mean of three rounds was registered as a unidimensional variable with an intraclass correlation coefficient (ICC) of 0.91[23]. Similarly, the “PPT index” (relative PPT value) was calculated in patients with CRC and shows the degree of sensitivity (%) [12]. This index is obtained by dividing the mean of each PPT point from patients by the mean of each PPT point in the healthy matched control group (HMCG). CRC patients with a higher PPT index were most consistent with HMCG. A difference of 20% between groups was considered clinically significant [24].

#### Self-report of spontaneous pain

Patients were asked to rate their pain intensity in the abdominal and lumbar areas using a horizontal visual analog scale (VAS) of 10 cm (cm), where 0 means “no pain” and 10 means “the worst pain.” This instrument has an ICC of 0.97 [25]. The cutoff scores for musculoskeletal pain were as follows: mild pain (0 to 3 points), moderate pain (3 to 6 points), and severe pain (> 6 points) [26].

### Abdominal isometric strength

Abdominal isometric strength was assessed using the trunk curl test to evaluate a possible alteration of the lumbopelvic functional stability. From a supine position with flexion of the knees and hips, patients flexed their trunk to separate the lower angle of the scapula from the stretcher and then maintained this position, with their arms extended without touching their knees as long as they could. Time was recorded up to a maximum of 90 s. This test has a high reliability (ICC > 0.97) [26].

### Muscle structure

Muscle images were captured using an ultrasound device (MyLab 25; Esaote Medical System, Genova, Italy) for the multifidus, transversus abdominis, and external and internal obliques (cm). A 12-MHz linear probe was used following a previous protocol [8]. The images were recollected at a depth of 5 cm with the patient lying on the stretcher during apnea. The reliability of the ultrasound images for multifidus (ICC = 0.55–0.86) and abdominal (ICC > 0.81) muscle thickness has been previously shown [27].

### Body composition and anthropometry

Body composition, musculoskeletal mass (kg), body fat (%), body mass index (BMI, kg/m^2^), and skeletal muscle mass index (musculoskeletal mass/height 2 (kg/m^2^)) were obtained using an InBody 720 tetrapolar eight-point tactile electrode system (Biospace Co., Ltd., Seoul, Korea). The patients were instructed to rest (no rigorous exercises in the previous 24 h) without a meal/water 3 h before measurement. The cutoff points related to a higher risk of CRC are a weight of 82 kg and a BMI of 31 kg/m^2^ [33]. The skeletal muscle mass index is based on physical disability risk and has been used as a usual cutoff to define moderate sarcopenia when it is between 8.51 and 10.75 kg/m^2^ (men) or 5.76 and 6.75 kg/m^2^ (women) [28].

Waist circumference (cm) was assessed using plastic tape at the end of exhalation at the midpoint between the lowest rib and iliac crest. A value of 87 cm is associated with a higher risk of CRC [29].

### Statistical analysis and data presentation

Analyses were performed using the SPSS statistical package for MacOS Sierra version 10.13 (IBM Corp. iReleased 2016, 24.0 version, Armonk, NY: IBM Corp.), with a level of significance of *p* < 0.05 and a 95% confidence interval (CI). The results are expressed as means (m) ± standard deviation (SD) for continuous variables or numbers (*n*) and percentages (%) for category variables. The Shapiro–Wilk test was used to check the normal distribution of the outcomes (*p* > 0.05). Analysis of variance (ANOVA) was performed to assess the similarity between groups for continuous variables related to demographic and clinical characteristics. The chi-squared (*χ*^2^) test was used for category variables. Three-way analysis of covariance (ANCOVA) was used to evaluate the between-group difference in outcomes with a normal distribution, with ages, stages, and cancer treatment as covariables. Post hoc analysis was performed with the Bonferroni test, and Cohen’s *d* effect size was calculated to quantify the between-group differences considered small (0.20), moderate (0.50), and large (0.80). The Kruskal–Wallis test was used when the outcomes did not reach normality, and post hoc comparisons were performed using the Dunn-Bonferroni post hoc method. Hedge’s effect size was calculated to quantify the between-group differences, which were considered small (0.20), moderate (0.50), and large (0.80). Additionally, Pearson’s test was used to analyze the bivariate correlation between the dominant lumbar side of the PPT and the remaining dependent outcomes in each group. A correlation from 0 to 0.25 indicates an absent or weak relationship, a correlation from 0.25 to 0.50 indicates a fair relationship, a correlation from 0.50 to 0.75 indicates a moderate to good relationship, and a correlation greater than 0.75 indicates a very good relationship [30]. Missing data were not included in the analysis.

## Results

Of the 239 screened patients, 110 were eligible to complete the assessment. The reasons for ineligibility included participation declination (*n* = 76), not meeting the inclusion or exclusion criteria (*n* = 46), and failure in the assessment instruments (*n* = 7). Finally, 29 patients (69.0% men) with an average age of 61.68 ± 12.78 years were included in the newly diagnosed group (NDG), 40 patients (65.0% men) with an average age of 60.80 ± 10.02 years were included in the post-treatment group (PTG), and 40 healthy matched people (52.5% men) with an average age of 59.54 ± 9.69 years were included in the HMCG. The demographic and clinical characteristics of each participant group are shown in Table 1.

**Table 1:** Demographic and clinical characteristics of the groups

		Newly diagnosed (*n* = 29)	Post-treatment (*n* = 40)	Healthy matched control (*n* = 40)	*p* value
Age (years) m ± SD		61.68 ± 12.78 1	60.80 ± 10.02	59.54 ± 9.69	0.712
Time since surgery (months) m ± SD		-	13.26 ± 8.76	-	-
Gender *n* (%)	Male	20 (69.0)	26 (65.0)	21 (52.5)	0.323
	Female	9 (31.0)	14 (35.0)	19 (47.5)	
Social situation *n* (%)	Single	2 (6.9)	3 (7.5)	2 (5.0)	0.101
	Married	22 (75.9)	34 (85.0)	30 (75.0)	
	Divorced	1 (3.4)	1 (2.5)	7 (17.5)	
	Widowed	4 (13.8)	2 (5.0)	1 (2.5)	
Smoking status *n* (%)	Never smoked	13 (44.8)	20 (50.0)	20 (50.0)	0.988
	Current smoker	3 (10.3)	4 (10.0)	4 (10.0)	
	Ex smoker	13 (44.8)	16 (40.0)	15 (37.5)	
Alcohol intake *n* (%)	Never	15 (51.7)	15 (37.5)	14 (35.0)	0.330
	Monthly	4 (13.8)	9 (22.5)	6 (15.0)	
	Weekly	2 (6.9)	9 (22.5)	11 (27.5)	
	Daily	8 (27.6)	7 (17.5)	7 (17.5)	
Physical activity level *n* (%)	< 10 MET/h w > 10 MET/h week	2 (7.7) 24 (92.3)	5 (12.5) 35 (87.5)	3 (8.3) 33 (91.7)	-
Cancer stage *n* (%)	I	5 (17.2)	0 (0.0)	-	-
	II	5 (17.2)	14 (35.0)	-	
	III	16 (55.2)	25 (62.5)	-	
Medical treatment	No treatment	29 (100)	8 (20.0)	40 (100)	-
	Radiotherapy	-	3 (7.5)	-	
	Chemotherapy	-	16 (40.0)	-	
	Radiotherapy and chemotherapy	-	13 (32.5)	-	

*p* values of between-group differences using ANOVA test for independent samples (continuous variables) and *X*^2^ analysis (categorical variables). *m*, mean; *n*, sample size; *SD*, standard deviation; % (percentage). *p < 0.05; **p < 0.001

### Pain

**Fig. 1:**
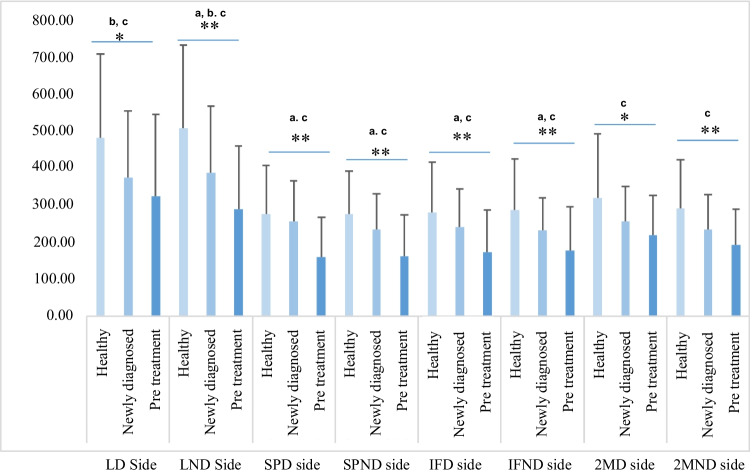
Pressure pain thresholds (kPa) between-groups differences. *p < 0.05; **p < 0.001; a—between newly diagnosed and post-treatment groups differences with Bonferroni post hoc; b—between newly diagnosed and healthy matched control groups differences with Bonferroni post hoc; c—between post-treatment and healthy matched control groups differences with Bonferroni post hoc. LD side, lumbar dominant side; LND side, lumbar nondominant side; SPD side, supraumbilical dominant side; SPND side, supraumbilical nondominant side; IFD side, infraumbilical dominant side; IFND side, infraumbilical nondominant side; 2MD side, second metacarpal dominant side; 2MND side, second metacarpal nondominant side

Figure 1 shows the PPT differences between groups. ANCOVA detected significant differences between groups at all PPT evaluation points: lumbar side (dominant; *F* = 5.4, *p* = 0.006; nondominant; *F* = 12.2, *p* < 0.001), supraumbilical side (dominant; *F* = 10.8, *p* < 0.001; nondominant; *F* = 10.8, *p* < 0.001), infraumbilical side (dominant; *F* = 7.8, *p* = 0.001; nondominant; *F* = 8.0 *p* = 0.001), and second metacarpal side (dominant; *F* = 5.5, *p* = 0.005; nondominant; *F* = 7.7, *p* = 0.001). The NDG and PTG registered lower values than the HMCG and were always lower in the PTG. The intergroup effect size between the NDG and PTG was large for the supraumbilical dominant side (*d* = 0.81; CI = 0.29, 1.32) and moderate for the lumbar nondominant side (*d* = 0.57; CI = 0.07, 1.06), supraumbilical nondominant side (*d* = 0.57; CI = 0.07, 1.07), infraumbilical dominant side (*d* = 0.61; CI = 0.11, 1.10), and infraumbilical nondominant side (*d* = 0.51; CI = 0.01, 0.99). The intergroup effect size between the NDG and HMCG was moderate for the dominant lumbar side (*d* = 0.52; CI = 0.01, 1.01) and nondominant lumbar side (*d* = 0.58; CI = 0.07, 1.07). The intergroup effect sizes between the PTG and HMCG were large for the nondominant lumbar side (*d* = 1.11; CI = 0.61, 1.57), supraumbilical points (dominant side: *d* = 0.98, CI = 0.49, 1.44; nondominant side: *d* = 1.01, CI = 0.52, 1.47), infraumbilical points (dominant side: *d* = 0.86, CI = 0.38, 1.32; nondominant side: *d* = 0.86, CI = 0.37, 1.32), and second metacarpal nondominant side (*d* = 0.86; CI = 0.38, 1.32) and moderate for the lumbar dominant side (*d* = 0.71; CI = 0.23, 1.16) and second metacarpal nondominant side (*d* = 0.70; CI = 0.22, 1.16). ANCOVA with cancer stage as a covariate influenced the results on the lumbar side (dominant *p* = 0.217; nondominant *p* = 0.631) and infraumbilical side (dominant *p* = 0.650; nondominant *p* = 0.128).

**Fig. 2:**
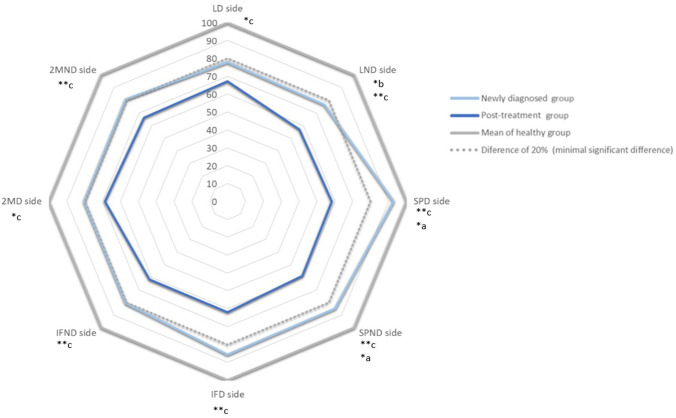
Pressure pain threshold index differences between newly diagnosed and post-treatment groups. *p < 0.05; **p < 0.001; a—between newly diagnosed and healthy matched control group differences with Bonferroni post hoc; b—between healthy matched control and post-treatment groups differences with Bonferroni post hoc; c—between newly diagnosed and post-treatment groups differences with Bonferroni post hoc; LD side, lumbar dominant side; LND side, lumbar nondominant side; SPD side, supraumbilical dominant side; SPND side, supraumbilical nondominant side; IFD side, infraumbilical dominant side; IFND side, infraumbilical nondominant side; 2MD side, second metacarpal dominant side; 2MND side, second metacarpal nondominant side

ANCOVA of the PPT index revealed the number of patients with significant clinical differences (> 20%) relative to the HMCG values for the lumbar dominant side (*n* = 14, 50.0% in the NDG; *n* = 27, 67.5% in the PTG), lumbar nondominant side (*n* = 16, 57.1% in the NDG; *n* = 29, 72.5% in the PTG), supraumbilical dominant side (*n* = 12, 41.3% in the NDG; *n* = 31, 77.5% in the PTG), supraumbilical nondominant side (*n* = 14, 50.0% in the NDG; *n* = 31, 77.5% in the PTG), infraumbilical dominant side (*n* = 13, 46.4% in the NDG; *n* = 30, 75.0% in the PTG), infraumbilical nondominant side (*n* = 14, 50.0% in the NDG; *n* = 26, 65.0% in the PTG), second metacarpal dominant side (*n* = 15, 53.5% in the NDG; *n* = 27, 67.5% in the PTG), and second metacarpal nondominant side (*n* = 17, 66.7% in the NDG; *n* = 27, 67.5% in the PTG). Figure 2 shows PPT index differences between NDG and PTG.

**Fig. 3:**
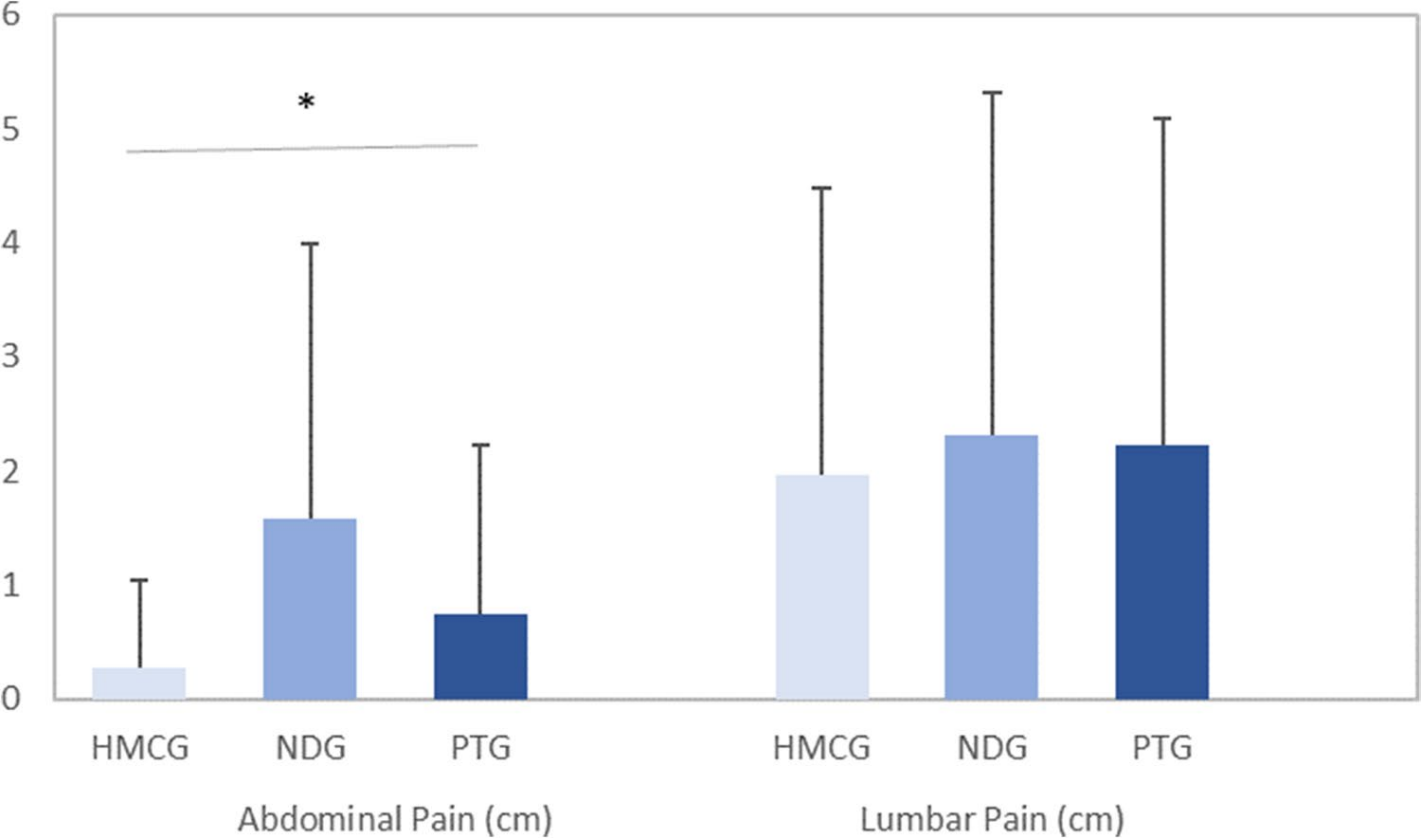
Between groups differences in VAS (cm) at abdominal and lumbar area. HMCG, healthy matched control group; NDG, newly diagnosed group; PTG, post-treatment group. *p < 0.05 with the Kruskal–Wallis test

The Kruskal–Wallis test of self-reported spontaneous pain revealed a significant difference between groups in abdominal pain (*p* = 0.006). Figure 3 shows differences in VAS (cm) at the abdominal and lumbar areas between groups. The post hoc analysis identified significant differences between the NDG and HMCG (*p* = 0.005). The intergroup effect size was moderate (*g* = 0.90; CI = 0.39, 1.40) between these groups (Fig. 3). No significant differences were found in lumbar pain (*p* = 0.920).

### Abdominal isometric strength

The Kruskal–Wallis test revealed a significant difference (*p* < 0.001) between groups for abdominal isometric strength, with lower values in the NDG and PTG than in the HMCG. Post hoc analysis identified significant differences between the NDG and HMCG (*p* = 0.011) and between the PTG and HMCG (*p* < 0.001). Table 2 shows comparisons between groups according to the abdominal isometric strength.

**Table 2:** Comparison between groups

		*m*		SD		95% CI			Effect size		95% CI			*p* value
Abdominal isometric strength														
*Trunk curl test* *(s)*	Newly diagnosed	42.11	34.86		28.85		55.37			0.60 b 1.49 c		0.10, 1.10 0.98, 1.99	< 0.001**¥ **b,c**	
	Post-treatment	24.89	10.92		21.39		28.38							
	Healthy matched control	62.65	34.15		51.26		74.04							
Muscle structure														
*Width lumbar multifidus (cm)*	Newly diagnosed	3.75		.68		3.49		4.01	0.84 b 0.88 c		0.33, 1.36 0.41, 1.36			< 0.001**¥ b, c
	Post-treatment	3.55		1.01		3.22		3.87						
	Healthy matched control	4.32		.65		4.10		4.54						
*Depth transversus abdominalis (cm)*	Newly diagnosed	0.38		0.22		0.30		0.47						0.339¥
	Post-treatment	0.35		0.15		0.30		0.40						
	Healthy matched control	0.39		0.12		0.35		0.43						
*Depth external oblique (cm)*	Newly diagnosed	0.63		0.31		0.51		0.75						0.102¥
	Post-treatment	0.53		0.29		0.44		0.62						
	Healthy matched control	0.65		0.33		0.54		0.76						
*Depth internal oblique (cm)*	Newly diagnosed	0.55		0.29		0.44		0.67						0.290¥
	Post-treatment	0.57		0.18		0.51		0.63						
	Healthy matched control	0.65		0.27		0.56		0.74						
Anthropometric and body composition outcomes														
*Musculoskeletal mass (kg)*	Newly diagnosed	30.63		6.63		28.05		33.20	0.62 a		0.12, 1.10			0.047* a
	Post-treatment	26.91		5.48		25.16		28.67						
	Healthy matched control	27.74		6.45		25.65		29.84						
*Body fat (%)*	Newly diagnosed	31.95		10.83		27.75		36.15						0.732
	Post-treatment	33.84		9.90		30.68		37.00						
	Healthy matched control	33.21		8.70		30.39		36.02						
*Body mass index (kg/m*^*2*^*)*	Newly diagnosed	29.90		5.32		27.84		31.97						0.327
	Post-treatment	28.34		5.01		26.74		29.94						
	Healthy matched control	28.07		5.27		26.36		29.78						
*Weight (kg)*	Newly diagnosed	80.78		17.33		74.05		87.50						0.279
	Post-treatment	75.63		12.59		71.60		79.66						
	Healthy matched control	75.52		14.73		70.74		80.29						
*Skeletal muscle index (kg/m*^*2*^*)*	Newly diagnosed	11.31		2.17		10.47		12.15	0.71 a		0.21, 1.20			< 0.05*¥ a
	Post-treatment	9.95		1.43		9.50		10.42						
	Healthy matched control	10.21		1.65		9.65		10,77						
*Waist circumference (cm)*	Newly diagnosed	101.00		12.70		95.98		106.02						0.137
	Post-treatment	101.90		12.43		97.92		105.87						
	Healthy matched control	96.16		12.58		91.62		100.69						

Between-group differences on *p* values using ANCOVA test for independent samples or Kruskal–Wallis test (¥). *m*, mean; *SD*, standard deviation; *p < 0.05; **p < 0.001; a, between newly diagnosed and post-treatment groups differences with Bonferroni post hoc; b, between newly diagnosed and healthy matched control groups differences with Bonferroni post hoc; c, between post-treatment and healthy matched control groups differences with Bonferroni post hoc

### Muscle structure

The Kruskal–Wallis test showed a significant difference in the width of the lumbar multifidus (*p* < 0.002) between groups, with lower values in the NDG and PTG than in the HMCG. Post hoc analysis identified significant differences between the NDG and HMCG (*p* = 0.011) and between the PTG and HMCG (*p* = 0.004). Table 2 shows the comparison between groups according to muscle structure.

### Body composition and anthropometric outcomes

ANCOVA of musculoskeletal mass data revealed a significant difference between groups (*F* = 3.14; *p* = 0.047), with lower values in the PTG than in the NDG and HMCG. Bonferroni post hoc analysis identified significant differences between the NDG and PTG (*p* = 0.014; CI = 0.76, 6.65). Additionally, the Kruskal–Wallis test showed a significant difference between groups for the skeletal muscle mass index (*p* = 0.038). Post hoc analysis identified significant differences between the NDG and PTG (*p* = 0.042). No significant differences were found for the remaining variables. Table 2 shows comparisons between groups according to body composition and anthropometric outcomes.

### Correlations

**Fig. 4:**
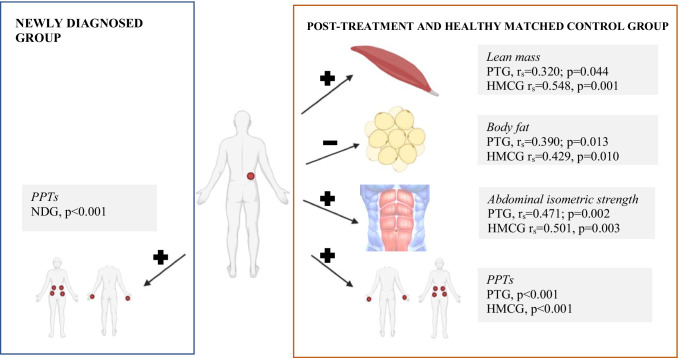
Bivariate correlation between lumbar dominant side of pressure pain threshold and the rest of dependent outcomes in each group. Created in BioRender.com. + positive correlation; − negative correlation. LDS, lumbar dominant side; NDG, newly diagnosed group; PPTs, pressure pain thresholds; PTG, post-treatment group; HMCG, healthy matched control group

In all groups, Pearson’s test showed a significant positive association (*p* < 0.001) between the dominant lumbar side point and remaining PPT points. In the PTG and HMCG, positive associations were observed between the dominant lumbar side points and abdominal isometric strength (*rs* = 0.471 and *p* = 0.002 in the PTG; *rs* = 0.501 and *p* = 0.003 in the HMCG) and musculoskeletal mass (*rs* = 0.320 and *p* = 0.044 in the PTG; *rs* = 0.548 and *p* = 0.001 in the HMCG). Additionally, negative associations were observed between the dominant lumbar side points and body fat (*rs* =  *− *0.390 and *p* = 0.013 in the PTG; *rs* =  *− *0.429 and *p* = 0.010 in the HMCG). Figure 4 shows a schematic representation of the bivariate correlation between the lumbar dominant side of the PPT and remaining dependent outcomes in each group.

## Discussion

We found that CP is already present in CRC patients at diagnosis prior to treatment. The analysis detected a threshold reduction in most PPT points, lower values in the PPT index, higher self-reported abdominal pain, and lower abdominal strength in newly diagnosed patients, with even more marked results in post-treatment patients, where lower lean mass and skeletal muscle index values were also found compared with those in the healthy matched controls.

Curiously, our findings of a reduction in PPT suggest that from the moment of diagnosis, patients with CRC had CP, indicating the possible onset of central sensitization without the presence of some of the factors that may enhance CP in the post-treatment group. Additionally, 1 of 2 patients in the NDG showed a minimal clinical difference (> 20%) in the PPT index compared with that in the HMCG. There are studies that address the issue of pain in newly diagnosed cancer patients, although their cohort is only partially treatment-naïve and it does not focus on patients with CRC. In the study by Ger et al. [31], a Taiwanese cohort of patients newly diagnosed with several types of cancer, including CRC, was analyzed. They found that 38% (*n* = 113) of the patients presented CP, and that only in 8% of those cases was due to cancer treatment. Also, they found that, among other reasons, pain prevalence correlated with patient socioeconomic characteristics (i.e., lower medical insurance coverage) and pain severity with a more advanced stage of the disease and previous inadequate pain management. In another study by Kelsen et al. [32], they analyzed data from newly diagnosed (64%), and just after their first chemotherapy (36%) patients with pancreas cancer. They found that there was a percentage having none (37%), mild (34%), or moderate-severe (29%) pain. Also, that their cohort presented less pain among the preoperative patients, but also that there was a correlation between depressive symptoms and pain (which 38% of the cohort presented). These results could show the influence from both physiological and psychosocial dimensions of pain [33], which are sometimes present at diagnosis.

Additionally, the isometric strength values were 30% lower in the NDG and almost 60% lower in the PTG than in the HMCG, findings that are consistent with other study findings from our research group on PTG patients [34, 35]. The lower abdominal strength in NDG patients was a negative finding and shows the possible loss of muscle strength that often accompanies chronic pain [36]. Furthermore, the lumbopelvic area is the central area of the body where muscle chains are located [37, 38]. Functional alteration of the area could be related to a greater possibility of sacral fractures [39], joint instabilities [40], and low back pain [41, 42]. Additionally, previous evidence has shown that NDG early-stage patients with CRC already show muscle dysfunction, a phenomenon considered undetected in clinical practice but that shows a strong association with vital clinical end points, including survival and treatment toxicity [43]. Such findings could be used to start programs focused on strength exercises from diagnosis to try to mitigate the detrimental effects of future treatments on muscle strength.

Related to general muscle mass, the skeletal muscle mass index indicated that only the PTG showed moderate sarcopenia, a prevalent problem in patients with cancer because it involves a higher risk of developing immediate postoperative complications and decreased tolerance to chemoradiotherapy because of side effects [44]. However, in the muscles around the tumor, both the NDG and PTG presented a width reduction (with 13.19% less lumbar multifidus width in the NDG and 17.82% in the PTG) compared with the HMCG, a finding that is consistent with previous findings in patients with CRC [15, 45]. This early impact in muscle close to the tumor location could be caused by tumor inflammation-released cytokines [46]. Additionally, multifidus reduction may be related to overall survival [47], and its dysfunction is strongly associated with chronic low back pain [48].

Correlation analysis revealed that the PTG and HMCG were unexpectedly similar, with reductions in the dominant lumbar side PPT correlated with the remaining PPT points, lower values of isometric abdominal strength, lean mass, and higher body fat in both groups. Better muscle function may mitigate pain perception [49, 50]; additionally, a lower PPT is related to an excessive fat percentage, which is also associated with body biomechanical/structural changes, increased inflammatory mediators, mood disturbance, poor sleep, and lifestyle issues [21], which may explain our findings. In the NDG, these correlations did not appear except for among the PPT points, and our algometry data in the NDG showed data dispersion. Therefore, we supposed that the wide range of variable responses might be due to the impact of the diagnosis. These findings highlight the importance of considering body composition, specifically increasing muscle and decreasing adipose tissue, in the pain management of these patients because it may indirectly affect their pain. In the case of newly diagnosed patients, body composition could help prevent this situation; however, additional studies are needed to clarify these findings.

Some limitations of our study should be noted. First, not all the factors that influence the development of central sensitization from the biopsychosocial perspective were analyzed in these patients; secondly, analyses with different groups limit the results, and no longitudinal changes could be studied; also, the study did not examine the presence of background pain or record any analgesic treatment; therefore, these characteristics were not established as inclusion criteria to establish a representative sample of patients with CRC.

This study also presents some strengths. Widespread pain, which is a crucial objective measure, was addressed. Also, this work attempts to respond to the limitations of a previous study in which prospective data from patients with CRC was needed to be obtained upon diagnosis [8]. Moreover, it highlights the deterioration of the health status at the time of diagnosis, thus reinforcing the need for multidisciplinary interventions that are necessary and must include, in addition to multimodal physical exercise interventions (endurance, resistance, strength, motor control, and flexibility, among others), educational, nutritional, and psychosocial support interventions [51].

## Conclusion

Before the start of cancer treatment, NDG patients with CRC show signs of primary hyperalgesia, central sensitization, and deterioration in physical condition and body composition. Such symptoms appear to be further aggravated following cancer treatment. Hence, addressing the health status of these patients at diagnosis is crucial.

## Data Availability

Data will be available upon request from the corresponding author.
